# Heterogeneous responses and cross reactivity between the major peanut allergens Ara h 1, 2,3 and 6 in a mouse model for peanut allergy

**DOI:** 10.1186/s13601-015-0056-9

**Published:** 2015-03-23

**Authors:** Joost J Smit, Maarten T Pennings, Karina Willemsen, Manon van Roest, Els van Hoffen, Raymond H Pieters

**Affiliations:** Immunotoxicology group, Institute for Risk Assessment Sciences, Utrecht University, Utrecht, The Netherlands; Utrecht Centre for Food Allergy, Utrecht, The Netherlands; Utrecht University Medical Center, Utrecht, The Netherlands; Current affiliation: NIZO food research BV, Ede, The Netherlands; Current affiliation: HU University of Applied Sciences, Utrecht, The Netherlands

**Keywords:** Cross-reactivity, IgE, Peanut allergy, Peanut proteins, T cell, Mouse model, Sensitization, Anaphylaxis

## Abstract

**Background:**

The relative contribution and the relation between individual peanut allergens in peanut allergic responses is still matter of debate. We determined the individual contribution of peanut proteins to B, T cell and allergic effector responses in a mouse model for peanut allergy.

**Methods:**

Mice were immunized and challenged by oral gavage with peanut protein extract or isolated allergens Ara h 1, 2, 3 and 6 followed by assessment of food allergic manifestations. In addition, T cell responses to the individual proteins were measured by an *in vitro* dendritic cell-T cell assay.

**Results:**

Sensitization with the individual peanut proteins elicited IgE responses with specificity to the allergen used as expected. However, cross reactivity among Ara h 1, 2, 3 and 6 was observed. T cell re-stimulations with peanut extract and individual peanut proteins also showed cross reactivity between Ara h 1, 2, 3 and 6. Despite the cross reactivity at the IgE level, only Ara h 2 and 6 were able to elicit mast cell degranulation after an oral challenge. However, after systemic challenge, Ara h 1, 2 and 6 and to lesser extent Ara h 3 were able to elicit anaphylactic responses.

**Conclusions:**

Ara h 1, 2, 3 and 6 sensitize via the intra-gastric route, but differ in their capacity to cause allergic effector responses. Interestingly, extensive cross reactivity at T cell and antibody level is observed among Ara h 1, 2, 3 and 6, which may have important implications for the diagnosis and therapy of peanut allergy. Awareness about the relative contribution of individual peanut allergens and cross reactivity between these allergens is of importance for current research in diagnostics and therapeutics for and the mechanism of peanut allergy.

## Background

Sensitization to peanut is characterized by the presence of IgE to a number of peanut allergens. Up till now, thirteen peanut allergens, designated Ara h 1–13, are recognized by the WHO/IUS allergen nomenclature subcommittee [1]. Literature suggests that the major allergens in peanut are Ara h 1, 2, 3, and 6, as defined by frequent and prominent IgE binding from patient sera [2-4]. Ara h 1 is a member of the 7/8 S globulin (vicilin) family of seed storage proteins belonging to the cupin superfamily. Ara h 2 and Ara h 6 are members of the 2S albumins (conglutinins) belonging to the prolamin superfamily and are 59% homologous to each other. Importantly, the C terminus of Ara h 2 has some homology with other peanut proteins, specifically with Ara h 6 [5]. Ara h 3 is a 11S globulin (legumins/glycinins) that belongs to the cupin superfamily [2,6].

Importantly, mono-sensitization to a single peanut allergen is rare. Moreover, as extensively reviewed in [7,8], cross reactivity between peanut proteins and tree nut proteins and other allergens are common, even among different protein families. However, clinical studies on cross-reactivity at IgE level between peanut proteins themselves are limited to one study [9] and data on cross reactivity at T cell level are completely lacking. Therefore, we studied 1) the potency of peanut extract (PE) and individual native (not recombinant) peanut proteins Ara h 1, 2, 3 and 6 to induce food allergic manifestations and 2) possible cross reactivity of IgE antibodies and T-cells between Ara h 1, 2, 3 and 6 proteins. For this purpose, we used a well-established mouse model, which has been widely used for many years and has been shown to mimic the clinical and immunological characteristics of human peanut allergy [10-13]. The advantage of these models is the generation of mono-sensitized individual mice, essential for the study of possible cross reactivity. For this purpose, we used well-defined, highly purified, native peanut proteins, which have been used in several previous mouse and human studies [3,4,14,15]. In this model, we observed extensive cross reactivity among peanut proteins at both T cell and IgE level in our models of peanut allergy. In addition, we confirmed that Ara h 2 and 6 are the main cause of effector responses such as mast cell degranulation and anaphylaxis.

Awareness about the relative contribution of individual peanut allergens and cross reactivity between the major peanut proteins is of importance for current research in the fields of diagnostics and therapeutics (including immunotherapy) and for our complete understanding of the mechanism of peanut allergy.

## Materials and methods

### Mice

Five-week-old specific pathogen-free female C3H/HeOuJ mice were purchased from Charles River (France) and housed under specific pathogen-free conditions within the animal care facility at the Utrecht University. Experiments were approved by the Animal Experiments Committee of the Utrecht University.

### Peanut, Ara h 1, 2, 3 and 6 sensitization and challenge

Raw peanuts were kindly provided by Intersnack BV (the Netherlands) and peanut extract (PE) was prepared as previously described [16]. Importantly, we used the same Ara h 1, 2, 3 and 6 preparations used and described before, with a purity of 95-99% as determined by SDS-PAGE electrophoresis and coomassie brilliant blue staining [3,4,15]. Lipopolysaccharide content, as determined by a limulus amoebocyte lysate assay (Cambrex Bio Science, Walkersville, MD, USA), of the peanut allergens was below 0.001 EU/mg protein. All proteins were dissolved and dosed in PBS. Cholera toxin (CT) was obtained from List Biological Laboratories, Inc. (CA, USA). To elicit oral sensitization to peanut proteins, 8 mice were intragastrically (i.g.) dosed by gavage with 6 mg PE or 250 μg Ara h 1, 2, 3, or 6 plus 15 μg CT per mouse for three consecutive days, and this was repeated every week for four weeks (exposure on days 0, 1, 2, 7, 14, 21 and 28). Control groups received PBS plus 15 μg CT. Mice received an i.g. challenge dose of 12 mg PE or 500 μg Ara h 1, 2, 3, or 6 on day 35, were bled after 30 minutes and sacrificed one day later. In separate experiments, 8 mice were i.g. sensitized with PE as described above, and challenged i.p. with 500 μg Ara h 1, 2, 3, or 6. After this systemic challenge, body temperature was measured by means of rectal thermometry every 10–20 minutes for 90 minutes after challenge. In addition, clinical symptoms were scored using a scoring system, as used before [17].

### Measurement of PE, Ara h 1, 2, 3, and 6-specific antibodies, MMCP-I and histamine in blood

PE, Ara h 1, 2, 3, and 6-specific IgE in serum obtained on day 28 was analyzed by ELISA as described previously [14,18]. This protocol was amended: a positive pool serum derived from PE/alum-sensitized mice was used as reference value to calculate arbitrary units (AU). For detection of specific IgE, PE, Ara h 1, 2, 3 and/or 6 was coupled to DIG. This coupling was performed according to the manufacturer’s instructions (Roche, The Netherlands) and coupled proteins were separated on a sephadex G-25 column and labeling efficiency was determined spectrophotometrically at 280 nm. In addition, plasma was collected within 45 minutes after oral challenge, and MMCP-I was determined in this plasma using a ELISA kit according to instructions of the manufacturer (eBioscience, Austria). Histamine was measured in plasma using a commercial EIA assay (Immunotech, France).

### Cell culture and cytokine measurement

Spleen single cell suspensions (2.5×10^6^/ml) were incubated in the presence or absence of PE, Ara h 1, 2, 3 or 6 (100 μg/ml) for 96 h at 37°C in complete RPMI1640 with 10% FCS. Culture supernatants were harvested and stored at −20°C until analysis. Levels of IL-5 and IL-13 in culture supernatants were determined by sandwich ELISA according to the instructions of the manufacturers (eBioscience, Austria). The detection limit is 5 pg/ml for both ELISA’s.

### Dendritic cell/T cell assay

This assay was used as described before [13]. In short, 2–3 C3H/HeOuJ mice were immunized with 100 μg PE, Ara h 1, 2, 3 or 6 bound to imject-alum (Pierce, USA) on day 0 and 14. After 29 days, the CD4+ T cells were isolated from spleen single cell suspensions using magnetic beads (Stemcell Technologies, France). Bone marrow cells from C3H/HeOuJ mice was cultured for 6 days with GM-CSF, after which the bone marrow-derived dendritic cells (DC) were pulsed overnight with 50 μg/ml PE Ara h 1, 2, 3 or 6. The CD4+ T cells were incubated for 72 h with protein-pulsed dendritic cells. Hereafter, IL-5 and IL-13 production was analyzed by sandwich ELISA (eBioscience, Austria).

### Statistical analysis

Data are presented as means ± standard error of the mean (SEM) and analyzed using GraphPad Prism software. Antibody, MMCP-I and cytokine levels were logarithmic transformed followed by a one-way ANOVA and Bonferroni as a post-hoc test. Temperature curves were statistically analyzed using a repeated measures ANOVA and clinical scores were statistically analyzed by the Kruskall-Wallis test.

## Results

### PE, Ara h 1, 2, 3, or 6-specific antibody and T cell responses in mice

**Figure 1:**
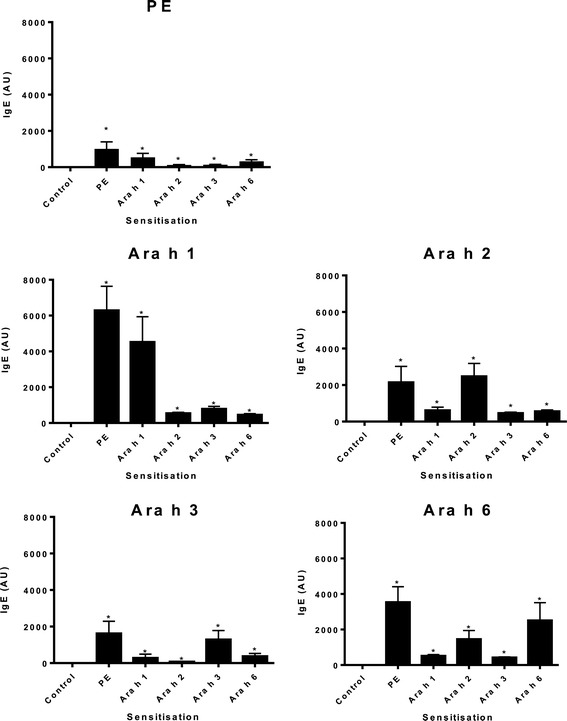
**IgE responses after intragastric sensitization.** C3H/HeOuJ mice were intragastrically exposed to PBS, (control) PE, or Ara h 1, 2, 3, or 6 and CT during a 4 week period, as described in material and methods. Graphs depict serum levels of PE, Ara h 1, 2, 3, or 6 specific IgE in arbitrary units (AU) at day 36. Data are represented as mean ± SEM of 8 mice. *: p < 0.01 compared to control.

Peanut allergen-specific IgE responses were compared in mice intragastrically exposed to PE, Ara h 1, 2, 3 or 6 with CT. Sensitization with PE induced Ara h 1, 2, 3 and 6 specific IgE (Figure 1). Sensitization with the individual peanut proteins led to the induction of IgE antibody responses with specificity to the allergen used (Figure 1). In addition, although generally at levels lower than the sensitizing allergen, extensive cross reactivity was observed between peanut proteins, since Ara h 1, 2, 3 and 6 specific IgE antibodies were detected in all sensitized groups (Figure 1).

**Figure 2:**
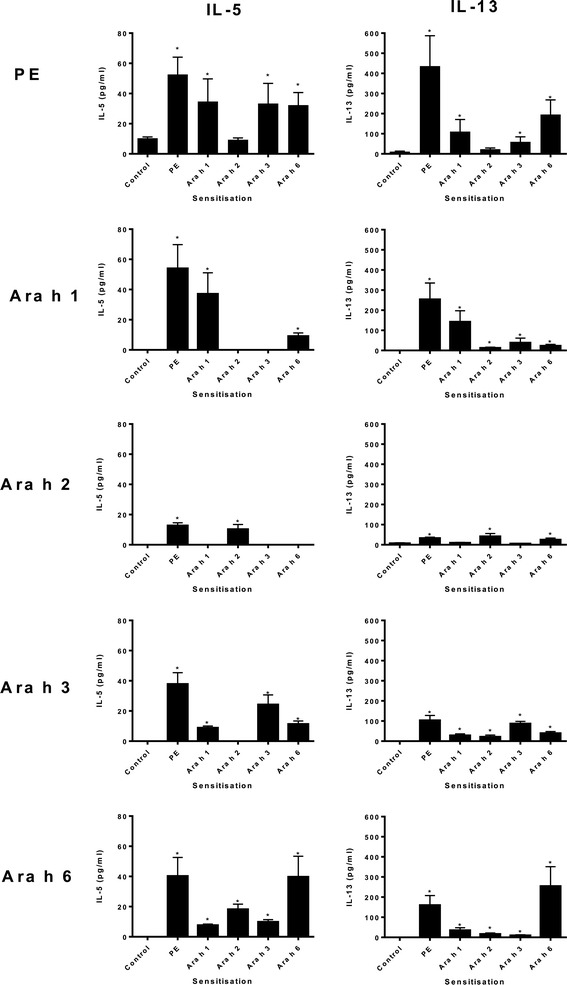
**Splenic T cell responses after intragastric sensitization.** C3H/HeOuJ mice were intragastrically exposed to PBS (control), PE, or Ara h 1, 2, 3, or 6 and CT during a 4 week period, as described in material and methods. Spleens were isolated and stimulated with medium alone or PE, or Ara h 1, 2, 3, or 6. Graphs depict levels of IL-5 and IL-13 after stimulation. Data are represented as mean ± SEM of 8 mice. *: p < 0.05 compared to control.

Next, spleen cells of mice sensitized with PE, Ara h 1, 2, 3, or 6 were restimulated with these respective proteins. Sensitization with PE, followed by restimulation with PE, Ara h 1, 2, 3, or 6 led to induction of the Th2 cytokines IL-5, IL-13 (Figure 2). In addition, PE restimulation of spleens derived from Ara h 1, 3, or 6 sensitized mice induced the same cytokine response. Restimulation with Ara h 2, however, led to lower Th2 cytokine responses in all groups. Furthermore, restimulation with the individual peanut proteins led to the induction of Th2 responses not only in mice of the same sensitizing allergen, but also in mice sensitized with one of the other peanut allergens. This cross reactivity was observed in Ara h 1, 3 and 6 sensitized mice for both IL-5 and IL-13. Despite that responses were generally lower, IL-13 could be measured in Ara h 2-sensitized mice after restimulation with Ara h 1, 3 and 6. IL-5 was measured in small amounts after restimulation with Ara h 2 and 6 in Ara h 2-sensitized mice. Results of both specific IgE and cytokine production *in vivo* are summarized in Table 1.

**Table 1:** **Summary of PE, Ara h 1, 2, 3 and 6 specific IgE and T cell responses** ***in vivo*** **(Figures** **1** **and** **2** **)**

**Specific IgE**					
→ IgE					
↓ sensitization	PE	Ara h 1	Ara h 2	Ara h 3	Ara h 6
PE	+++	+++	++	++	++
Ara h 1	++	+++	+	+	+
Ara h 2	+	+	++	+	++
Ara h 3	+	+	+	++	+
Ara h 6	++	+	+	+	++
**Spleen T cell responses (IL-5 and IL-13)**					
→ restim.					
↓ sensitization	PE	Ara h 1	Ara h 2	Ara h 3	Ara h 6
PE	+++	++	+/−	++	++
Ara h 1	++	++	-	+	+
Ara h 2	+	-, +(IL-13)	+/−	+	+
Ara h 3	+	-, +(IL-13)	-	++	+
Ara h 6	++	+	-, +/−(IL-13)	+	++

### DC-T cell responses to PE, Ara h 1, 2, 3 and 6

**Figure 3:**
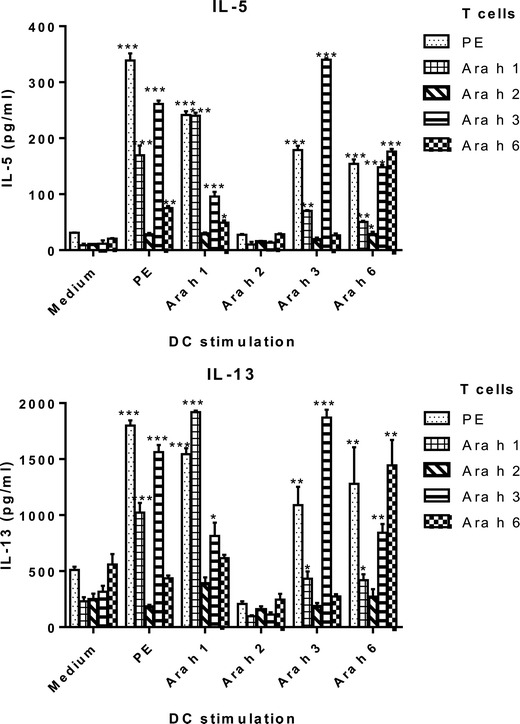
**Dendritic cell -T cell responses to peanut proteins ex vivo.** Bone marrow derived dendritic cells were pulsed overnight with PE, Ara h 1, 2, 3 or 6. Hereafter, these dendritic cells were incubated for 72 h with splenic CD4+ T cells derived from mice immunized with PE, Ara h 1, 2, 3 or 6. Graphs depict levels of IL-5 and IL-13 after stimulation. Shown is a representative of 3 experiments and data are represented as mean ± SEM of quadruplicates. *: p < 0.05, **: p < 0.01 or ***: p < 0.001 compared to medium control.

In order to further investigate whether the observed cross reactivity originated at the level of DC-T cell interaction, a sensitive and specific (using purified CD4+ T cells) *in vitro* DC-T cell assay was used. Mice were immunized intraperitoneally by Ara h 1, 2, 3, or 6/alum injection, which led to comparable levels of specific IgG (data not shown) demonstrating that T cell responses to Ara h 1, 2, 3, and 6 did develop. Importantly, no IgG cross-reactivity was observed between mice sensitized for Ara h 1, 2, 3 and 6/alum (data not shown). Restimulation of CD4+ T cells derived from Ara h 1, 2, 3, or 6-immunized mice with PE, Ara h 1, 2, 3, or 6-pulsed DC resulted in production of IL-5 and IL-13 (Figure 3). Both Th2 cytokines were induced most effectively in T cells derived from Ara h 1, 3, or 6 sensitized mice after pulsing of DC with PE or the corresponding allergen. In this assay, pulsing with Ara h 2 did not lead to cytokine production from CD4+ T cells. Importantly, also in this assay, extensive cross reactivity was observed among Ara h 1, 3 and 6, as summarized in Table 2.

**Table 2:** **Summary of PE, Ara h 1, 2, 3 and 6 specific T cell responses after pulsing with dendritic cells (DC)** ***in vitro*** **(Figure** **3** **)**

**→ DC**					
**↓ T cell**	**PE**	**Ara h 1**	**Ara h 2**	**Ara h 3**	**Ara h 6**
PE	+++	+++	-	++	+++
Ara h 1	++	+++	-	+	+
Ara h 2	-	-	-	-	-
Ara h 3	+++	+	-	+++	++
Ara h 6	-, +(IL-5)	-, +(IL-5)	-	-	+++

### Mast cell degranulation and systemic anaphylaxis

**Figure 4:**
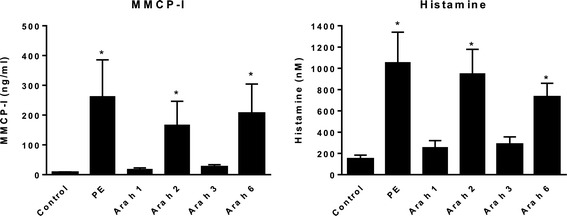
**Mucosal mast cell degranulation after intragastric challenge with Ara h 1, 2, 3, or 6.** C3H/HeOuJ mice were intragastrically exposed to PBS, (control) PE, or Ara h 1, 2, 3, or 6 and CT during a 4 week period, as described in material and methods. On day 35, mice received an i.g. challenge of PE or Ara h 1, 2, 3, or 6, depending on the protein used during sensitization, followed by blood withdrawal. Graphs depict the level of MMCP-I and histamine in plasma. Data are represented as mean ± SEM of 8 mice. *: p < 0.05 compared to control.

Subsequently, it was assessed whether the antibody and T cell responses to Ara h 1, 2, 3 and 6 would lead to clinical manifestations of food allergy as well. First, mucosal mast cell degranulation was measured after intra-gastric challenge with PE, Ara h 1, 2, 3 and 6 in correspondingly sensitized mice by measuring mMCP-1 and histamine in plasma. Interestingly, only PE, Ara h 2 and 6 were able to induce mucosal mast cell degranulation and histamine release in sensitized mice (Figure 4).

**Figure 5:**
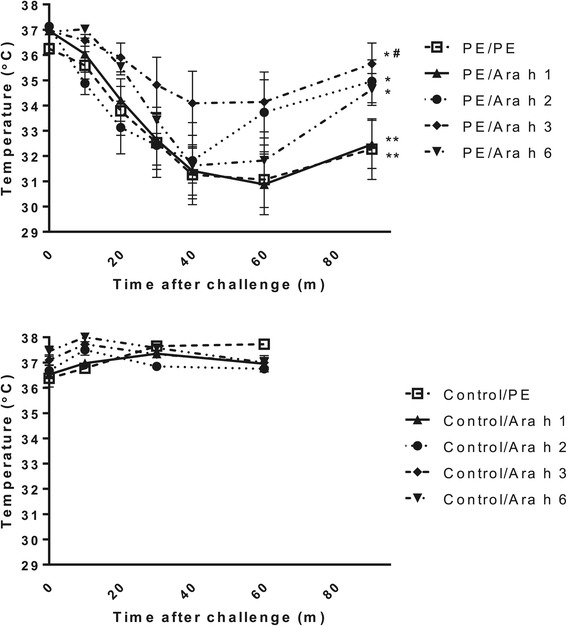
**Anaphylaxis after systemic challenge with Ara h 1, 2, 3, or 6.** C3H/HeOuJ mice were intragastrically exposed to PBS, (control) or PE and CT during a 4 week period, as described in material and methods. On day 35, mice received a systemic i.p. challenge with PE or Ara h 1, 2, 3, or 6, directly followed by measurement of rectal temperature at indicated time points after challenge. Upper panel shows the PE sensitized mice, lower panel shows the not sensitized control mice. Data are represented as mean ± SEM of 8 mice. *: p < 0.05 or **: p < 0.01 compared to control. #: p < 0.05 compared to PE challenged mice.

Intragastric challenge with PE or Ara h 1, 2, 3 or 6 did not lead to anaphylactic symptoms in sensitized mice, as also described before [11]. Therefore, control or PE-sensitized mice were intraperitoneal challenged with PE or Ara h 1, 2, 3, 6 and changes in temperature were measured. An anaphylactic response, reflected by a strong drop in temperature, was observed in PE-sensitized mice after challenge with PE, Ara h 1, 2, 3 and 6 (Figure 5). However, this drop in temperature was significantly less in Ara h 3 challenged mice compared to other sensitized groups. The systemic injection of control, naive mice with peanut or Ara h 1, 2, 3 and 6 did not lead to any anaphylactic responses (Figure 5).

## Discussion

In the present study, we demonstrated that Ara h 1, 2, 3 and 6 are individually able to sensitize mice via the intra-gastric route, as measured by induction of IgE and Th2 type cytokines. Despite the sensitizing capacity of Ara h 1, 2, 3 and 6, only Ara h 2 and 6 were able to elicit mast cell degranulation after intra-gastric challenge. These data append earlier studies from our lab and others which showed that sensitization with peanut extract induces Ara h 1, 2, 3, 6 specific responses [14] and that Ara h 2 and 6 are the main cause of effector responses [5,19], including anaphylaxis, in mouse models. In addition, human clinical and *in vitro* data showed that although the large majority of patients show Ara h 1, 2, 3 and 6 specific IgE responses, Ara h 2 and 6 are the main cause of mast cell or basophil effector responses [3,4,9]. After systemic challenge however, Ara h 1, 2 and 6 and to lesser extent Ara h 3 were able to elicit anaphylactic responses. Differences in studies may have been caused by the use of native purified proteins in our studies instead of depleted peanut extracts but possibly also by cross reactivity among these proteins, as shown in the present study and discussed above.

Interestingly, restimulation of splenocytes or CD4+ T cells from mono-sensitized mice in our studies showed similar T cell responses compared to the restimulation of short-term Ara h 1, 2, 3 or 6 specific T cell lines from young allergic patients [20]. In both mouse and human assays, Ara h 2 is a poor stimulator of T cell responses, while the other peanut proteins induce significant Th2 type responses. However, others have found T cell responses after stimulation with Ara h 2 [21,22] or Ara h 2/6 [23]. Differences in Ara h 2 preparations (which are made using different denaturing agents for instance), T cell selection, age of the patients and use of short-term versus long-term T cell lines may account for these differences. We cannot explain the lower or even lack of T cell stimulation by Ara h 2 *ex vivo* and *in vitro* in our studies. In contrast, Ara h 6, which is 60% homologous to Ara h 2 [2] was able to stimulate T cells in our and the human studies [20]. Since mice and human do develop T cell-dependent IgE responses to Ara h 2 *in vivo*, we believe that the lack of T cell stimulation by Ara h 2 may be caused by the high stability and lack of degradation of this protein by DC *in vitro* [24].

Importantly, extensive cross reactivity among Ara h 1, 2, 3 and 6 at the level of IgE was observed, although at a level always lower than for the specific sensitizing allergen. Moreover, splenic T cell re-stimulations, *ex vivo* and *in vitro*, also showed cross reactivity between Ara h 1, 3 and 6. This cross reactivity as observed is dependent on purity and possible contamination of Ara h 1, 2, 3 and 6. The purity of the proteins used in this study has been described in several previous publications and varies around 99% [4]. This small percentage of possible contamination is unlikely to have caused the level of cross reactivity observed. In both the animal dosing (<2.5 μg) and detection ELISA (<0.1 μg/ml) the contamination would have been too low to cause contaminative responses almost to the same level as the original protein. Importantly, we did not observe IgG cross-reactivity between mice intraperitoneal sensitized for Ara h 1, 2, 3 in the presence of alum. This indicates that although minute contaminants of other proteins may still be present in individual allergen preparations, these are not responsible for our findings.

The observed cross reactivity is not completely unexpected. Previously, it has been shown that Ara h 1, 2 and 3 display a high extent of cross-reactivity at the IgE level, using peanut allergic patient sera [9]. This may be surprising given the fact that Ara h 1 and 3 belong to the cupin protein family and Ara h 2 and 6 to the 2S albumins, without any clear structural or alignment sequence similarities. Nonetheless, previous studies showed the occurrence of similar epitopes among peanut allergens by IgE binding [9] or biochemical sequence similarities [25]. Functional cross reactivity between proteins of different protein families is rare, but occurs. For instance, it was shown that peanut Ara h 2 was cross-reactive with the walnut allergen Jug r 2 belonging to the different vicilin protein family [26]. However, despite the observed extensive cross reactivity among the peanut allergens in our studies, distinct Ara h 1, 2, 3 and 6-specific epitopes are present which are not shared among the other peanut allergens. This was shown in the already mentioned human IgE inhibition studies [9], but also in our experiments, since the cross reactivity resulted usually in a lower level of IgE or Th2 cytokine production, when compared to the sensitizing allergen. In addition, the observed cross reactivity may even extend to the level of clinical responses, when comparing the occurrence of anaphylaxis between all groups.

Cross reactivity between Ara h 1, 2, 3 and 6 may have several implications. First, cross reactivity may explain the multiple sensitization patterns to peanut. Most peanut allergic patients develop IgE to multiple peanut proteins, mostly to Ara h 1, 2, 3, and 6 [3,27]. In addition, both the increased diversity of epitopes recognized and higher level of IgE binding correlated with the severity of the clinical reaction to peanut [27,28]. Unfortunately, due to the limited amount of mouse serum an extensive mapping of epitope binding could not be performed in our studies. Second, the observed cross reactivity, especially at the level of IgE, may have consequences for the diagnostics of Ara h 1, 2, 3, 6-specific responses. Seemingly specific peanut allergen responses in peanut allergic patients may be caused by cross reactivity in the used assay with other peanut proteins. Third, cross reactivity may be used in (immune-) therapy for peanut allergy. Mouse studies already suggested that immunotherapy with Ara h 2/6 was able to inhibit clinical responses after whole peanut extract challenge in peanut sensitized mice [19]. These clinical responses are dependent only on Ara h 2/6, but we can envision that immunotherapy with Ara h 2/6 could not only be effective in lowering responses to this particular allergen but also to other cross reactive peanut proteins.

## Conclusions

In summary, we showed that cross reactivity among the main peanut allergens may exist at the IgE and T cell level, and that DC- T cell interactions underlie this cross reactivity. In addition, we showed that, depending on the route of provocation, Ara h 2 and 6 are the peanut allergens causing clinical responses in our model. These data complement the very limited human clinical studies on peanut allergen cross reactivity, but more research is needed on where and how the immune system recognizes and processes these peanut allergens. This study provides substantial new information on the capacity of peanut allergens to cause sensitization and food allergy, and may have important consequences for diagnostics and immunotherapy of peanut allergy.

## Abbreviations

PE: Peanut extract
DC: Dendritic cell
CT: Cholera toxin
AU: Arbitrary units
